# Diagnostic Accuracy of CT-Guided Transthoracic Needle Biopsy for Solitary Pulmonary Nodules

**DOI:** 10.1371/journal.pone.0131373

**Published:** 2015-06-25

**Authors:** Wen Yang, Wenkui Sun, Qian Li, Yanwen Yao, Tangfeng Lv, Junli Zeng, Wenjun Liang, Xiaojun Zhou, Yong Song

**Affiliations:** 1 Department of Respiratory Medicine, Jinling Hospital, Nanjing University, School of Medicine, Nanjing, China; 2 Department of pathology, Jinling Hospital, Nanjing University, School of Medicine, Nanjing, China; University of Utah Health Sciences Center, UNITED STATES

## Abstract

To evaluate the diagnostic accuracy of computed tomography (CT)-guided percutaneous lung biopsy for solitary pulmonary nodules. Three hundred and eleven patients (211 males and 100 females), with a mean age of 59.6 years (range, 19–87 years), who were diagnosed with solitary pulmonary nodules and underwent CT-guided percutaneous transthoracic needle biopsy between January 2008 and January 2014 were reviewed. All patients were confirmed by surgery or the clinical course. The overall diagnostic accuracy and incidence of complications were calculated, and the factors influencing these were statistically evaluated and compared. Specimens were successfully obtained from all 311 patients. A total of 217 and 94 cases were found to be malignant and benign lesions, respectively, by biopsy. Two hundred and twenty-five (72.3%) carcinomas, 78 (25.1%) benign lesions, and 8 (2.6%) inconclusive lesions were confirmed by surgery and the clinical course. The diagnostic accuracy, sensitivity, and specificity of CT-guided percutaneous transthoracic needle biopsy were 92.9%, 95.3%, and 95.7%, respectively. The incidences of pneumothorax and self-limiting bleeding were 17.7% and 11.6%, respectively. Taking account of all evidence, CT-guided percutaneous lung biopsy for solitary pulmonary nodules is an efficient, and safe diagnostic method associated with few complications.

## Introduction

With improved awareness of public health and the recent advances of various imaging technologies, the detection rate of solitary pulmonary nodules (SPNs) is continuously increasing, with the reported detection rate currently being 8–51% [1]. An SPN is classically defined as a single, discrete, round or oval opacity, less than or equal to 3 cm in diameter that is completely surrounded by lung parenchyma, does not touch the hilum or mediastinum, and is not associated with adenopathy, atelectasis, or other lung lesions [2]. Accurate diagnosis of SPNs has always been difficult, and represents a substantial problem in chest imaging diagnoses. Preoperative differentiation of benign and malignant SPNs is critical to reducing the rate of unnecessary operations on benign SPNs, and is hence of utmost importance. For years, computed tomography-guided percutaneous transthoracic needle biopsy (CT-PTNB) has represented a major approach for the diagnosis and differential diagnosis of pulmonary masses, owing to its simplicity and minimal invasiveness. However, recently, CT-PTNB has been challenged by new technologies with outstanding safety results, such as electromagnetic navigation bronchoscopy (ENB) and endobronchial ultrasonography (EBUS). Thus, we cannot help but wonder if, with the constant development of new technologies, CT-guided PTNB will ultimately be replaced? To evaluate the diagnostic accuracy of CT-guided PTNB for SPN, we conducted 311 SPN patients and analysed the associated complications, and discuss the diagnostic value and safety issues of CT-PTNB in the present study.

## Methods

### Patients

**Fig 1:**
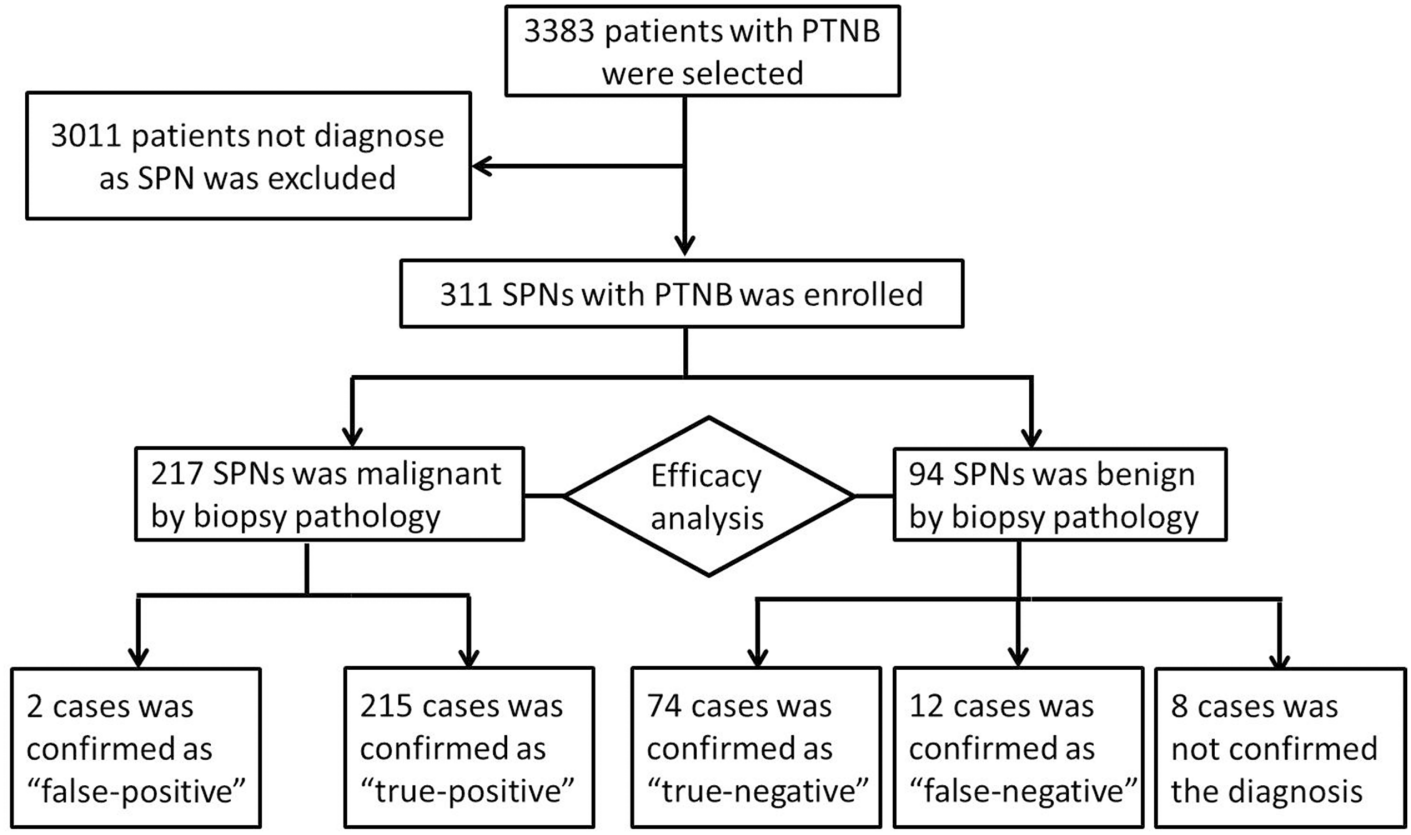
Flow diagram of the eligible patients and the enrolling process of the study.

Between January 2004 and January 2014, 3383 patients underwent percutaneous CT-guided lung biopsy at our hospital. Of these, 311 (9.2%) consecutive cases identified as SPNs were enrolled. The diameters of all nodules were measured using lung window settings. All patients had undergone diagnostic chest CT, either in our hospital or at another hospital, before the biopsy (Fig 1).

### Ethics Statement

This study was carried out in accordance with the Declaration of Helsinki and local institutional ethical and legal requirements. The study was approved by the Medical Ethics Committee of Jinling Hospital, Nanjing, China. The written informed consent was received from all participants or statutory guardians before study enrollment.

### Procedure

All biopsies were performed by three physicians experienced in pneumology and radiology. A commercially available CT scanner was used for the biopsy. The patients underwent CT in the prone, supine, or lateral position based on the shortest distance from the lesion to the body surface. Images were obtained from the region of interest by using a section thickness of 5 mm and were viewed by using lung window settings. One experienced physician performed all biopsies.

After local anaesthesia with 2% lidocaine from the skin to pleura, a coaxial 18-gauge needle (Lot Number, REXK0682; Bard Peripheral Vascular, Inc., Tempe, AZ), (15 cm or 9 cm in length) was inserted under intermittent CT guidance with its trajectory pointing toward the lung lesion. The initial puncture was performed without penetrating the pleura. CT images were obtained to assess the position of the biopsy needle. If the nodule was on the extended path of the needle track, the biopsy procedure was continued. When the nodule was penetrated, the needle tip was checked and a specimen was obtained. This procedure was typically performed once, and occasionally performed twice. The patients were instructed to hold their breath during the CT scanning and the biopsy process. The resected specimen was placed in 10% formaldehyde for pathological examination.

The needle path length was defined as the length between the skin surface and the centre of the solitary pulmonary nodule. In the lung, the needle path length was defined as the length between the pleura and the centre of the solitary pulmonary nodule. The distance to the pleura was defined as the distance from the pleura of the puncture to the centre of the solitary pulmonary nodule. The needle-pleural angle was defined as the angle formed between the biopsy needle and the tangent to the pleura.

After removal of the biopsy needle, completion images were obtained to detect any post-biopsy complication, such as pneumothorax and haemorrhage. The patients were requested to stay supine for at least 6 hours and were only allowed to get off the bed 24 hours later. The placement of a chest tube was considered in the event a patient became symptomatic or a large (>30%) pneumothorax was found.

### Diagnostic criteria

Ture positive: I)Patients whose biopsy histopathological findings showed malignancy and who received surgical resection were considered as malignant. II) Positive findings at biopsy histopathology were considered to be true-positive when biopsy of another site revealed cancer with the same histologic characteristics, or when the lesion increased in size and other proven metastases were found, or when the tumour size was reduced after radiotherapy, chemotherapy, or targeted therapy.

Ture negative: I)Patients whose biopsy histopathological findings showed a benign lesion and received surgical resection were considered as benign. II)Patients whose biopsy histopathological findings showed a benign lesion that had shrunk, disappeared, or remained unchanged after at least 1 year follow-up.

False positive: I)Patients whose biopsy histopathological findings showed malignancy and surgical resection yielded a benign diagnosis. II) The lesions subsequently disappeared or decreased in size or remained stable on the follow-up CT for at least 1year.

False negative: I)Patients whose biopsy histopathological findings showed benign and surgical resection yielded a malignancy diagnosis. II) The lesions increased in size or other proven metastases were diagnosed.

Inconclusive diagnose: patients who lost of follow-up or the follow-up time being less than 1 year were defined.

### Statistical analysis

Statistical analyses of the data were performed using SPSS version 21 (SPSS Inc., Chicago, IL). The diagnostic sensitivity, specificity, positive predictive value, and negative predictive value of CT-guided PTNB were calculated using the standard definitions. True-positive and true-negative cases were considered diagnosed cases. Falsepositive, false-negative and inconclusive diagnose cases were considered nondiagnosed cases. The diagnostic accuracy, and pneumothorax rate were statistically compared for each influencing factor using bivariate logistic regression analyses and the Chi-Square test, with P values <0.05 considered statistically significant.

## Results

### Diagnostic accuracy

Three hundred and eleven consecutive patients fulfilled the diagnostic criteria of SPN and were enrolled in this study. The patient characteristics are shown in Table 1. Specimen samples were successfully obtained from 311 SPNs (Table 2).

**Table 1:** Patient demographics and nodule characteristics (N = 311).

Characteristic	N	%
Age (years)		
Range	19–87	
Mean	59.6	
Sex		
Male	211	67.8
Female	100	32.2
Size of Pulmonary Nodule		
>10 mm	3	1
>10 mm and ≤20 mm	152	48.8
>20 mm and ≤20 mm	156	50.2
Location of Pulmonary Nodule		
Peripheral	221	71.1
Central	90	28.9
Left upper lobe	76	24.4
Left lower lobe	65	20.9
Right upper lobe	99	31.8
Right middle lobe	14	45
Right lower lobe	57	18.3
Smoker		
Yes	153	49.2
No	158	50.8
History of cancer		
Yes	19	6.1
No	292	93.9

**Table 2:** Percutaneous lung biopsy details.

Characteristic	N
Needle path length in lung (mm)	
≤10	3
11–20	93
21–30	81
31–40	55
41–50	39
51–60	19
>60	21
Position of patients	
Prone	155
Supine	129
Lateral	27
No. of punctures	
1	274
2	35
3	2

Of the 311 SPNs, 217 and 94 cases were found to be malignant and benign lesions, respectively, by biopsy. Of these, 215 cases diagnosed as malignant and 74 diagnosed as benign were found to be true-positive and true-negative cases, respectively. Two malignant and 12 benign SPNs were false-positive and false-negative cases, respectively. The revising diagnoses were established at surgery (n = 9), by sputum cytology (n = 1), or by follow-up examination (n = 2). The remaining 8 patients were diagnosed as ‘inconclusive’, owing to being lost to follow-up (n = 4) or the follow-up time being less than 1 year (n = 4).

The final diagnoses were established as follows: 225 (72.3%) lesions were malignant (158 adenocarcinoma, 34 squamous cell carcinoma, 4 undifferentiated non-small cell carcinomas, 6 small cell carcinomas, 4 large cell carcinomas, 1 pulmonary sarcoma, 2 adenosquamous carcinomas, 2 adenocarcinoma in situ, and 14 metastatic carcinomas), 78 (25.1%) lesions were benign (2 sclerosing haemangiomas, 24 tuberculosis, 45 organised pneumonia, 2 pulmonary hamartoma, 2 pulmonary aspergillosis, and 1 vasculitide), and 8 lesions (2.6%) were inconclusive (Table 3).

**Table 3:** Final diagnoses (N = 311).

Final Diagnosis	N	%
*Malignant*	225	72.3
Lung adenocarcinoma	158	50.9
Lung squamous cell carcinoma	34	11
Lung large cell carcinoma	4	1.3
Small cell lung carcinoma	6	1.9
Undifferentiated lung carcinoma	4	1.3
Metastatic carcinomas	14	4.5
Pulmonary sarcoma	1	0.3
Lung adenosquamous carcinoma	2	0.6
Lung adenocarcinoma in situ	2	0.6
*Benign*	78	25.1
Sclerosing haemangioma	2	0.6
Tuberculosis	24	7.7
Organised pneumonia	45	14.5
Pulmonary hamartoma	2	0.6
Pulmonary aspergillosis	4	1.3
Vasculitides	1	0.3
*Inconclusive*	8	2.6
*Total*	311	100.0

* Inconclusive diagnose cases were considered nondiagnosed cases in calculation of diagnostic accuracy.

The overall diagnostic yield of CT-PTNB for SPN was 92.9%. The sensitivity, specificity, and negative and positive predicted values for malignancy were 95.3%, 95.7%, 78.6%, and 99.2%, respectively.

The results of the logistic regression analyses of factors associated with the diagnostic accuracy of CT-PTNB are shown in Table 4. The diagnostic accuracy was significantly affected by the lesion size (*P* = 0.003) and the number of punctures (*P* = 0.006).

**Table 4:** Diagnostic accuracy and pneumothorax rates according to clinicodemographic characteristics.

**Characteristic**	Diagnostic accuracy		Pneumothorax rate	
	OR (95%CI)	*P* vaule	OR (95%CI)	*P* vaule
Age (years)	1.859 (0.666~5.193)	0.237	2.401 (1.278~4.479)	**0.006**
Sex	1.675 (0.574~4.889)	0.345	0.922 (0.463~1.837)	0.818
Size of Nodule	1.161 (1.051~1.283)	**0.003**	1 (0.936~1.068)	0.988
Needle path length	0.986 (0.959~1.014)	0.317	1.031 (1.011~1.051)	**0.007**
No. of punctures	0.267 (0.105~0.681)	**0.006**	4.166 (1.955~8.7)	**0.001**

*OR*: *odds ratio*.

Further analysis of the 200 non-small cell lung cancer patients showed that stage IA, IIIA, IIIB, and IV tumours accounted for 100 (50%), 27 (13.5%), 9 (4.5%) and 64 (32%) cases, respectively.

### Complications of CT-PTNB biopsy

Pneumothorax occurred in 55 (17.7%) patients, with 3 cases (0.9% of all patients and 5.5% of the pneumothorax patients) requiring thoracotomy tube placement. The remaining 52 patients improved without any treatment. Higher rates of pneumothorax were encountered in patients older (*P* = 0.006), in cases where a long needle path length in the lung was used (*P* = 0.007), in patients who underwent CT-PTNB in the lateral position (*P* = 0.034), and in patients who underwent repeated puncture (*P* = 0.001) (Table 4).

Pulmonary haemorrhage was found in 36 (11.6%) patients, and haemoptysis occurred in 11 (3.5%) patients. In all patients, the bleeding stopped when treated with hematischesis drugs. Twenty-five (8%) patients showed some blood in the sputum after CT-PTNB.

## Discussion

SPN is a relatively common clinical disease. With the recent advances in imaging technologies, the incidence of SPN has been steadily increasing each year, and the diagnosis of SPN has become an important task for clinicians and radiologists. The malignant rate of SPN in clinical cases has been reported to range 10–68% [3]. Previously, the diagnosis of benign or malignant SPN was determined mainly by the imaging features. The majority of previous studies have suggested that lobulation, spiculation, and adjacent pleural indentation of the nodule were risk factors of malignancy, whereas partial calcification was indicative of benign nodules. In addition, more studies later reported that the risk of malignancy may also be closely related to the patients’ gender, age, smoking history, and history of malignant tumours [4, 5].

In our study, 303 cases of SPN were diagnosed, and 74.3% of these were found to be malignant. Further analysis showed that among 200 cases of non-small cell lung cancer, 63.5% of stage IA and IIIA patients showed indication for operation, suggesting that timely diagnosis of SPN is critical to ensure early diagnosis and prompt treatment of lung cancer.

Obtaining a tissue biopsy for pathological examination is key to ensuring prompt and appropriate diagnosis and treatment of SPNs. Haaga and Alfidi reported the first case of CT-PTNB in 1976 [6], and, since then, CT-PTNB has been constantly developed and is currently widely employed as a routine diagnostic technique for SPN due to its several advantages, such as a high true-positive rate, minimal invasiveness, and low cost. Out of the 311 SPN patients in our study, there were 2 false positive cases, 12 false negative cases, and 8 undiagnosed cases, resulting in a diagnostic accuracy of CT-PTNB of 92.9%. Logistic regression analyses revealed that nodule size and the number of needle punctures could affect the diagnostic accuracy, whereas the depth of needle puncture and the patients’ body position did not. To a certain degree, the number of needle punctures depended on the size and location of the nodule. For most of the nodules, we could obtain a sufficient specimen by only one biopsy. However, when the nodule was too small or difficult to access, and if the first specimen was not sufficient for pathological examination, a second biopsy was needed in some cases.

In the past decade, new techniques have emerged that offer guidance through the tracheobronchial tree during bronchoscopy, which help reach and biopsy the nodule, such as ENB and EBUS [7, 8]. Owing to the development and advantages of these new technologies, we cannot help but wonder whether CT-PTNB will ultimately be replaced?

The diagnostic accuracy of peripheral pulmonary nodules by EBUS and ENB has been reported as 46–86.2% [9–11] and 62.5–76.9%, respectively [12, 13]. Steinfort et al.[14] comprehensively analysed 1420 EBUS biopsies of peripheral pulmonary tumours from 16 studies, and reported a sensitivity of 0.73 (95% confidence interval [CI], 0.70–0.76). In addition, a meta-analysis of 15 studies involving 1033 patients with SPN by Gex et al.[15] showed that the diagnostic accuracy of pulmonary nodules with ENB was 73.9% (95% CI, 68.0–79.2). Accordingly, our results show that, in some instances, CT-PTNB can be at least as accurate as ENB and EBUS. Although it should be noted that some, more central lung lesions, are more amenable to EBUS-directed sampling.

Regarding the safety of PTNB, the major complications are known to be pneumothorax and pulmonary haemorrhage, with reported incidence rates of 10–40% and 26–33%, respectively [16]. In our study, out of all 311 SPN patients, there were 55 cases of pneumothorax (17.7%), 2 cases for whom thoracentesis was performed and one case for whom tube drainage was performed, while the rest were mild pneumothorax cases that recovered after self-absorption. Moreover, there were 36 cases of self-limiting pulmonary haemorrhage (11.6%) without fatal adverse reactions. Correlation analysis revealed that the incidence of pneumothorax was closely related to age (*P* = 0.006), number of needle punctures (*P* = 0.001), the patients’ body position during CT-PTNB (*P* = 0.034), and the depth of needle puncture inside the lung (*P* = 0.007), with older age, multiple needle punctures, and deeper needle punctures being major risk factors for pneumothorax. In addition, as mentioned above, we noticed that the patients’ position during needle puncture also greatly correlated with the risk of pneumothorax. The incidence of pneumothorax was higher when the patients were biopsied in the lateral position, as compared to in the supine and prone positions. We speculate that this is likely owing to the fact that it was more difficult for the patient to hold still for a long time in the lateral position during the CT scanning and puncture, and that any movement/change of body position might lead to pneumothorax. Although the incidence rates of pneumothorax and pulmonary haemorrhage of CT-PTNB in this study were higher than that of bronchoscopic biopsy, the 55 cases of pneumothorax and 36 cases of haemorrhage were all relatively mild, and the patients recovered after treatment without any fatal complications.

In terms of cost, CT-PTNB at a 3 armour hospital in China costs approximately 1,500 RMB ($241), whereas EBUS costs about 3,500 RMB ($563), and ENB costs around 8,000 RMB ($1287). Therefore, CT-PTNB has a significant price advantage.

In the last decade, we carried out more than three thousand CT-PTNB procedures, which yielded a high diagnostic accuracy and no fatal adverse complications. Having strict selection criteria of the appropriate patients is one of the most important factors for achieving this high diagnostic accuracy and low rate of adverse reactions. For CT-PTNB, the risk of pneumothorax increases if the nodule is near the hilum or away from the surface, if the puncture path passes the pulmonary bulla, or if the patient’s lung function is compromised. On the other hand, the risk of haemorrhage is high if the nodule is located near large blood vessels, and other diagnostic methods should be considered in these cases.

In conclusion, CT-PTNB has several advantages, including a high diagnostic accuracy, low cost, and manageable adverse reactions. With proficient operating skills and precise positioning of the puncture, the diagnostic accuracy of CT-PTNB can be greatly improved, and its complications can be minimised. Thus, this conventional method is still useful for most SPN cases, as determined based on the SPN features; and development of new complementary technologies will likely enhance the usefulness of this diagnostic procedure even further.

## Supporting Information

S1 Checklist STARD Checklist. (PDF)

## References

[1] Wahidi MM, Govert JA, Goudar RK, Gould MK, McCrory DC. Evidence for the treatment of patients with pulmonary nodules: when is it lung cancer?: ACCP evidence-based clinical practice guidelines (2nd edition). Chest. 2007;132(3 Suppl):94S–107S. Epub 2007/10/06. 10.1378/chest.07-1352 . 17873163

[2] Ost D, Fein AM, Feinsilver SH. Clinical practice. The solitary pulmonary nodule. N Engl J Med. 2003;348(25):2535–42. Epub 2003/06/20. 10.1056/NEJMcp012290 . 12815140

[3] Ost D, Fein A. Evaluation and management of the solitary pulmonary nodule. Am J Respir Crit Care Med. 2000;162(3 Pt 1):782–7. Epub 2000/09/16. 10.1164/ajrccm.162.3.9812152 . 10988081

[4] Swensen SJ, Silverstein MD, Ilstrup DM, Schleck CD, Edell ES. The probability of malignancy in solitary pulmonary nodules. Application to small radiologically indeterminate nodules. Arch Intern Med. 1997;157(8):849–55. Epub 1997/04/28. . 9129544

[5] Gould MK, Ananth L, Barnett PG. A clinical model to estimate the pretest probability of lung cancer in patients with solitary pulmonary nodules. Chest. 2007;131(2):383–8. Epub 2007/02/14. 10.1378/chest.06-1261 17296637 PMC3008547

[6] Haaga JR, Alfidi RJ. Precise biopsy localization by computer tomography. Radiology. 1976;118(3):603–7. Epub 1976/03/01. 10.1148/118.3.603 . 1251009

[7] McNulty W, Cox G, Au-Yong I. Investigating the solitary pulmonary nodule. BMJ. 2012;344:e2759 Epub 2012/04/21. 10.1136/bmj.e2759 . 22517934

[8] Zhan P, Xie H, Xu C, Hao K, Hou Z, Song Y. Management strategy of solitary pulmonary nodules. J Thorac Dis. 2013;5(6):824–9. Epub 2014/01/11. 10.3978/j.issn.2072-1439.2013.12.13 24409361 PMC3886686

[9] Eberhardt R, Anantham D, Herth F, Feller-Kopman D, Ernst A. Electromagnetic navigation diagnostic bronchoscopy in peripheral lung lesions. Chest. 2007;131(6):1800–5. Epub 2007/04/03. 10.1378/chest.06-3016 . 17400670

[10] Yoshikawa M, Sukoh N, Yamazaki K, Kanazawa K, Fukumoto S, Harada M, et al Diagnostic value of endobronchial ultrasonography with a guide sheath for peripheral pulmonary lesions without X-ray fluoroscopy. Chest. 2007;131(6):1788–93. Epub 2007/06/15. 10.1378/chest.06-2506 . 17565021

[11] Eberhardt R, Ernst A, Herth FJ. Ultrasound-guided transbronchial biopsy of solitary pulmonary nodules less than 20 mm. Eur Respir J. 2009;34(6):1284–7. Epub 2009/05/23. 10.1183/09031936.00166708 . 19460785

[12] Makris D, Scherpereel A, Leroy S, Bouchindhomme B, Faivre JB, Remy J, et al Electromagnetic navigation diagnostic bronchoscopy for small peripheral lung lesions. Eur Respir J. 2007;29(6):1187–92. Epub 2007/03/16. 10.1183/09031936.00165306 . 17360724

[13] Lamprecht B, Porsch P, Pirich C, Studnicka M. Electromagnetic navigation bronchoscopy in combination with PET-CT and rapid on-site cytopathologic examination for diagnosis of peripheral lung lesions. Lung. 2009;187(1):55–9. Epub 2008/10/07. 10.1007/s00408-008-9120-8 . 18836886

[14] Steinfort DP, Khor YH, Manser RL, Irving LB. Radial probe endobronchial ultrasound for the diagnosis of peripheral lung cancer: systematic review and meta-analysis. Eur Respir J. 2011;37(4):902–10. Epub 2010/08/10. 10.1183/09031936.00075310 . 20693253

[15] Gex G, Pralong JA, Combescure C, Seijo L, Rochat T, Soccal PM. Diagnostic yield and safety of electromagnetic navigation bronchoscopy for lung nodules: a systematic review and meta-analysis. Respiration. 2014;87(2):165–76. Epub 2014/01/10. 10.1159/000355710 . 24401166

[16] Yeow KM, See LC, Lui KW, Lin MC, Tsao TC, Ng KF, et al Risk factors for pneumothorax and bleeding after CT-guided percutaneous coaxial cutting needle biopsy of lung lesions. J Vasc Interv Radiol. 2001;12(11):1305–12. Epub 2001/11/08. . 11698630 10.1016/s1051-0443(07)61556-5

